# Birth Weight Percentiles and Infant and Child Growth Dynamics

**DOI:** 10.1001/jamanetworkopen.2026.22218

**Published:** 2026-07-08

**Authors:** María Alejandra Hernandez, Richard M. A. Parker, Tim J. Cole, Izzuddin M. Aris, Henrique Barros, Johan G. Eriksson, Abby F. Fleisch, Barbara Heude, Yung Seng Lee, Zheyuan Li, Emily Oken, Susana Santos, Kok Hian Tan, Chloe Vainqueur, Tanja G. M. Vrijkotte, John Wright, Tiffany C. Yang, Fabian Yap, Siyu Zhou, Kate Tilling, Deborah A. Lawlor, Ahmed Elhakeem

**Affiliations:** 1 MRC Integrative Epidemiology Unit, University of Bristol, Bristol, United Kingdom; 2 Department of Population Health Sciences, Bristol Medical School, University of Bristol, Bristol, United Kingdom; 3 UCL Great Ormond Street Institute of Child Health, London, United Kingdom; 4 Department of Population Medicine, Harvard Medical School and Harvard Pilgrim Health Care Institute, Boston, Massachusetts; 5 EPIUnit ITR, Instituto de Saúde Pública da Universidade do Porto, Universidade do Porto, Porto, Portugal; 6 Yong Loo Lin School of Medicine, National University of Singapore, Singapore, Singapore; 7 Institute for Human Development and Potential, Agency for Science, Technology, and Research, Singapore, Singapore; 8 Center for Interdisciplinary Population & Health Research, MaineHealth Institute for Research, Westbrook, Maine; 9 Pediatric Endocrinology and Diabetes, Maine Medical Center, Portland; 10 Université de Paris, Inserm, INRAE, Centre for Research in Epidemiology and StatisticS (CRESS), Paris, France; 11 School of Mathematics and Statistics, Henan University, Kaifeng, Henan, China; 12 KK Women’s and Children’s Hospital, Singapore, Singapore; 13 Department of Public and Occupational Health, Amsterdam UMC, University of Amsterdam, Amsterdam, the Netherlands; 14 Amsterdam Public Health Research Institute, Amsterdam, the Netherlands; 15 Bradford Institute for Health Research, Bradford Teaching Hospitals Foundation Trust, Bradford, United Kingdom

## Abstract

**Importance:**

Infants classified as small or large for gestational age can have different growth patterns compared with appropriate-for-gestational age counterparts. The association of birth weight percentiles beyond conventional thresholds with early-life growth remains unknown.

**Objective:**

To quantify the association of birth weight percentile range with infant and child growth.

**Design, Setting, and Participants:**

This is a prospective cohort study of singletons born between 1991 and 2011 in 7 birth cohort studies in Europe, Singapore, and the US and followed up with repeated growth measurements for 10 years. Five European cohorts were used for discovery analysis, and the Singapore and US cohorts were used for replication analyses.

**Exposures:**

Birth weight percentiles standardized for sex and gestational age using the INTERGROWTH-21st standards and classified into 10 decile groups, with the middle (fifth and sixth decile groups) as the reference group.

**Main Outcomes and Measures:**

The primary outcomes were infant height (centimeters per month) and weight (grams per month) growth velocity at 1, 6, 12, 24 months; body mass index (BMI; calculated as weight in kilograms divided by height in meters squared); age (months or years) at infant BMI peak and childhood BMI rebound; and overweight or obesity at 10 years. Associations were examined using regression models adjusted for sex and birth cohort.

**Results:**

The discovery cohort included 36 018 children (mean [SD] gestational age at birth, 39.7 [1.8] weeks; 17 238 girls [48%]). Compared with the reference group, higher decile groups had lower early infant height velocity that reversed by 24 months, higher weight velocity from 6 to 24 months, higher and earlier peak BMI, higher rebound BMI, and increased risk of overweight or obesity at age 10 years. Lower decile groups showed the opposite patterns. For example, mean differences for infant peak BMI were −0.38 (95% CI, −0.43 to −0.33) for the second decile birth weight group and 0.33 (95% CI, 0.29 to 0.38) for the ninth decile birth weight group compared with the fifth to sixth decile birth weight group. Mean differences for age at peak BMI were 0.22 months (95% CI, 0.12 to 0.33 months) for the second decile birth weight group and −0.21 months (95% CI, −0.30 to −0.11 months) for the ninth decile birth weight group compared with the fifth to sixth decile birth weight groups. Risk ratios for overweight or obesity at 10 years were 0.86 (95% CI, 0.76 to 0.97) for the second decile birth weight group and 1.25 (95% CI, 1.13 to 1.38) for the ninth decile birth weight group. Birth weight was not associated with age at rebound BMI. Replication analyses (2517 children; mean [SD] gestational age at birth, 39.2 [1.8] weeks; 1191 girls [47%]) supported these findings. Associations were typically linear and similar in boys and girls. Deciles provided only modest estimation gains over conventional categories.

**Conclusions and Relevance:**

In this cohort study of 38 535 singletons, birth weight decile was associated with early-life growth patterns. Birth weight decile group may help identify high-risk children missed by conventional thresholds, although the benefit of analysis using decile group over traditional groups remains modest.

## Key Points

**Question:**

Is birth weight–for–gestational age decile group associated with infant and child growth features?

**Findings:**

In this multicohort study of 36 018 European-born singletons and 2517 singletons born in the US and Singapore, compared with the middle (fifth to sixth) reference decile group, higher birth weight decile groups had lower infant height velocity that reversed by 24 months, higher infant weight velocity, higher and earlier infant peak body mass index (BMI), higher childhood rebound BMI, and higher risk of overweight or obesity at 10 years. Lower birth weight groups showed the opposite patterns.

**Meaning:**

These findings suggest that birth weight decile group is a simple and robust marker of obesogenic growth patterns.

## Teaser

This cohort study examines the association of birth weight decile range with infant and child growth.

## Introduction

Infant and childhood growth patterns reflect developmental processes driving the risk of later obesity. For instance, rapid infant weight gain,^1^ higher childhood body mass index (BMI), including higher and earlier BMI rebound,^2^ and faster increase in childhood BMI^3^ are all associated with a higher risk of subsequent obesity. Examining factors associated with these growth patterns is, therefore, crucial for understanding the developmental origins of obesity, enabling earlier identification of at-risk children, and informing effective interventions to reduce obesity burden.^4,5^

Abnormal fetal growth is an important clinical indicator of neonatal and postnatal morbidity.^6^ Population-based birth weight–for–gestational age standards are widely used to classify infants as small for gestational age (SGA; <10th percentile) or large for gestational age (LGA; >90th percentile). SGA and LGA are considered proxies for fetal growth restriction and overgrowth, respectively, and are used to identify infants at increased risk for morbidity.^7,8^ Studies show that SGA and LGA offspring can have unique childhood growth patterns, distinct from infants born appropriate for gestational age (AGA).^9,10,11^ Reliance on SGA and/or LGA thresholds obscures variation across the birth weight spectrum and limits insight into how fetal growth relates to infant and childhood growth. Granular analyses could enhance understanding of biological pathways underlying obesity and uncover subtle yet clinically meaningful differences in growth, improving early identification of infants at risk of later obesity.^12,13,14^ Moreover, comparing cohorts born in different geographic regions can improve generalizability. The aim of this study was to quantify associations of birth weight percentiles with infant height and weight growth velocity and longitudinal growth, the magnitude and timing of infant BMI peak and childhood BMI rebound, and childhood overweight or obesity in a pooled cohort, and to replicate the findings in an independent cohort.

## Methods

### Cohort Studies

Prospective pregnancy and birth cohort studies were identified from the European Union Child Cohort Network^15,16^ and collaborating studies. Cohorts were eligible for this study if they collected data on gestational age at birth, birth weight, and sex and had at least 5 repeated measurements of length, height, and weight between ages 1 week and 10 years. From the eligible cohorts, children were selected if they were singletons with available data on sex, gestational age, birth weight, and at least 2 growth measurements.

Five European Union Child Cohort Network birth cohort studies from the Netherlands (Amsterdam Born Children and their Development^17^), UK (Avon Longitudinal Study of Parents and Children^18,19,20^ and Born in Bradford^21^), France (Etude des Déterminants pré et post natals précoces du développement psychomoteur et de la santé de l’Enfant),^22^ and Portugal (Generation XXI)^23^ met eligibility and were included (eMethods in Supplement 1). An additional birth cohort from the US (Project Viva)^24,25^ and one from Singapore (Growing Up in Singapore Toward Healthy Outcomes Study)^26^ were also included (eMethods in Supplement 1). Mothers were recruited during pregnancy (6 studies) or labor (Generation XXI). Data were collected from parents and offspring using questionnaires, medical and health records, and research clinics. To assess generalizability and transportability of findings across different geographic regions, European birth cohorts were combined and used as a discovery cohort, and the 2 cohorts born in the US and Singapore were combined and used as a replication cohort. Discovery cohort participants were born between 1991 and 2011, and replication cohort participants were born in 1999 and 2010. Both cohorts were followed up from birth for up to 10 years.

Each cohort study was reviewed and approved by an institutional or national ethics board, and all study participants gave informed consent or assent to participate in their respective cohorts and secondary analyses. Details on cohort-specific ethics approval and consent is provided in eMethods in Supplement 1. Additional consent was not obtained for the current study, which was approved by the ALSPAC Ethics and Law Committee. This study is reported in line with the Strengthening the Reporting of Observational Studies in Epidemiology (STROBE) reporting guidelines.

### Birth Weight Percentiles

Gestational age at birth and birth weight were retrieved from health records. Birth weight was standardized by sex and gestational age using the International Fetal and Newborn Growth Consortium for 21st Century (INTERGROWTH-21st) standards. The INTERGROWTH-21st standards were developed on more than 20 000 healthy live births from 8 countries (Brazil, Italy, Oman, United Kingdom, US, China, India, Kenya).^27^ For our main analysis, birth weight was categorized into 10 percentile groups based on the INTERGROWTH-21st cutoffs, which we refer to as decile groups throughout the article (decile 1, <10th percentile; decile 2, 10th to <20th percentiles; decile 3, 20th to <30th percentiles; decile 4, 30th to <40th percentiles; deciles 5 to 6, 40th to <60th percentiles; decile 7, 60th to <70th percentiles; decile 8, 70th to <80th percentiles; decile 9, 80th to 90th percentiles; and decile 10, >90th percentile). The 2 middle decile groups (fifth to sixth decile groups) were combined and used as the reference group. We did not exclude any preterm and postterm births to increase generalizability to the whole birth weight–for–gestational age range.

### Infant and Child Growth Outcomes

Repeated length, height, and weight measurements from 1 week to 10 years and ages of measurement were available from health records and research clinic assessments (eFigure 1 in Supplement 1). Data were cleaned to remove errors.^28^ BMI was calculated as weight in kilograms divided by height in meters squared. The median (IQR) number of measurements per child was 7 (5-13) in the discovery cohort and 15 (9-18) in the replication cohort. Height, weight, and BMI trajectories up to age 10 years were estimated using sex-stratified P-splines linear mixed effects models^29,30^ separately in the discovery and replication cohorts (eMethods in Supplement 1).

The fitted growth curves (eFigure 2 in Supplement 1) were used to estimate growth outcomes for subsequent analysis. Infant height and weight growth velocity at 1, 6, 12, and 24 months was estimated from the derivatives of the fitted height and weight trajectories. The fitted trajectories were also used to estimate height and weight at 1-month intervals from 1 to 60 months, which were then converted to height-for-age and weight-for-age *z* scores using the World Health Organization (WHO) Child Growth Standards^31^ to compare longitudinal growth patterns relative to standards and across height and weight. Derivatives of the fitted BMI trajectories were used to estimate magnitude (BMI) and age of infant BMI peak (highest BMI following its rapid rise after birth^32^) and childhood BMI rebound (nadir or lowest BMI after the infant peak marking the second BMI rise^33^). Prevalence of overweight or obesity at age 10 years was derived by applying the International Obesity Task Force age-specific and sex-specific cutoff points to estimated BMIs.^34^ Further details are shown in the eMethods in Supplement 1.

### Statistical Analysis

Mean differences in estimated infant growth velocity (at ages 1, 6, 12, and 24 months), BMI, and age at infant peak BMI and childhood rebound BMI for each birth weight decile group (vs decile group 5-6) were estimated using linear regression. Height-for-age and weight-for-age *z* score trajectories for each birth weight decile group from age 1 month to 5 years were estimated using linear mixed effects models. Modified Poisson regression models with robust SEs were used to estimate risk ratios for overweight or obesity at 10 years for each birth weight decile group (vs decile group 5-6). All models were fitted in male and female children combined, and separately in the discovery and replication cohorts, with adjustment made for sex and birth cohort. The *z* score trajectory models included a natural spline for age^35^ plus its interaction with birth weight decile. Metaregression was used to test for differences in estimates between the discovery and replication cohorts.

The following additional analyses were conducted in the discovery cohort. Sex differences were examined by fitting models with interactions between birth weight group and sex. To evaluate whether using birth weight decile groups offered estimation benefit over the 3 conventional groups (SGA, AGA, and LGA), we used coefficient of determination (*R*^2^) and area under receiver operating characteristics curve to compare the performance of 2 competing linear and Poisson models, respectively. We interrogated the nonlinear nature of associations by comparing models with continuous birth weight *z* score entered as linear term vs natural spline terms. The role of preterm and postterm birth^36^ was examined by repeating the main analysis after excluding births less than 37 weeks and greater than or equal to 42 weeks gestation. Because prenatal growth could plausibly influence postnatal growth,^37,38^ we explored robustness of results to measured confounders (ie, factors that could influence both prenatal and postnatal growth) by fitting models with further adjustment for pregnancy-related covariates (maternal parity, maternal ethnicity, and both parents’ BMI, age, smoking, and education). Missing data in covariates were imputed using multiple imputation, and results compared with complete case analysis.

Analysis was done using R statistical software version 4.5.2 (R Project for Statistical Computing). Further details are in the eMethods in Supplement 1.

## Results

### Participant Characteristics

**Figure 1.**
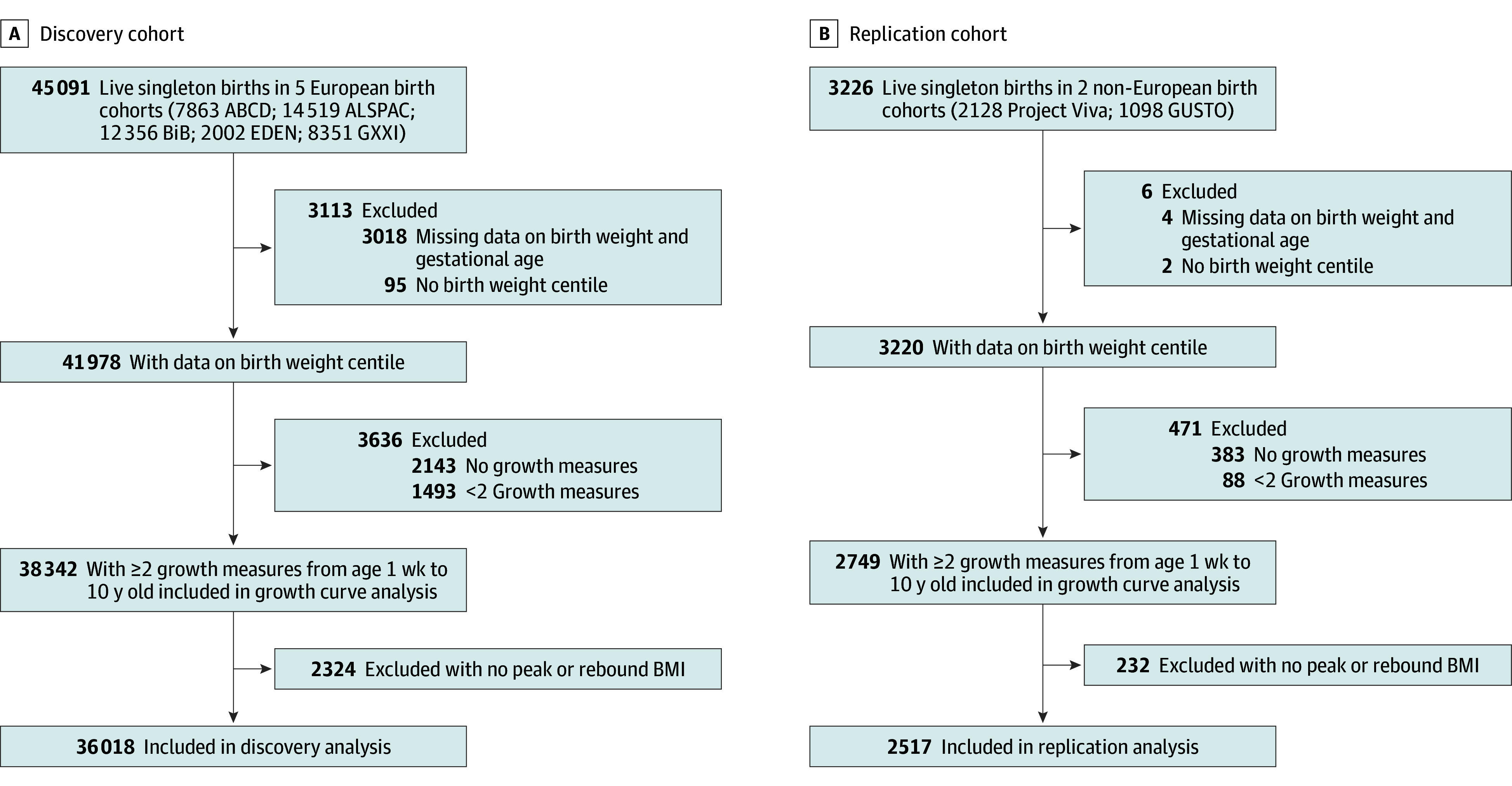
Study Flowchart ABCD indicates Amsterdam Born Children and their Development; ALSPAC, Avon Longitudinal Study of Parents and Children; BiB, Born in Bradford; BMI, body mass index; EDEN, Etude des Déterminants pré et post natals précoces du développement psychomoteur et de la santé de l’Enfant; GUSTO, Growing Up in Singapore Toward Healthy Outcomes; and GXXI, Generation XXI.

A total of 36 018 births (mean [SD] gestational age at birth, 39.7 [1.8] weeks; 17 238 girls [48%]) were included in the discovery cohort, and 2517 births (mean [SD] gestational age at birth, 39.2 [1.8] weeks; 1191 girls [47%]) were included in the replication cohort (Figure 1). Discovery cohort participants were born in 1991 to 2011 in Amsterdam, the Netherlands (5678 children [16%]); Bristol, UK (12 277 children [34%]); Bradford, UK (10 512 children [29%]); Nancy and Poitiers, France (1698 children [5%]); and Porto, Portugal (5853 children [16%]). Replication cohort participants were born in 1999 to 2010 in Massachusetts, US (1607 children [64%]), and Singapore (910 children [36%]).

Birth characteristics and growth outcomes are summarized in the Table for the discovery and replication cohorts, and in eTable 1 in Supplement 1 for each subcohort. Mean birth weight, gestational age, and birth weight percentile were similar in both cohorts, including a similar range of gestational age (25 to 43 weeks). In both cohorts, the lowest 4 decile groups included fewer than 10% of study participants, whereas the top 2 groups had more than 10%. Infant growth velocity and peak BMI, and the BMI and age at rebound BMI were broadly similar in both cohorts, but the discovery cohort had older age at peak BMI and lower prevalence of overweight or obesity at 10 years. Correlations between continuous growth outcomes were mostly low to moderate and consistent across cohorts (eFigure 3 in Supplement 1), suggesting they capture partially distinct aspects of growth.

**Table.** Birth Characteristics and Infant and Child Growth Outcomes in the Discovery and Replication Cohorts

Characteristic	Participants, No. (%)					
	Discovery cohort^a^			Replication cohort^b^		
	All (N = 36 018)	Boys (n = 18 780)	Girls (n = 17 238)	All (N = 2517)	Boys (n = 1326)	Girls (n = 1191)
Birth weight, mean (SD), g^c^	3320 (545)	3375 (560)	3260 (521)	3337 (564)	3398 (578)	3268 (541)
Gestational age, mean (SD), wk	39.7 (1.8)	39.7 (1.8)	39.8 (1.7)	39.2 (1.8)	39.2 (1.8)	39.2 (1.7)
Birth weight percentile, mean (SD), %	53.0 (29.5)	53.2 (29.6)	52.8 (29.4)	58.2 (28.9)	58.4 (28.9)	57.8 (28.9)
Birth weight decile group^d^						
First	3436 (9.5)	1783 (9.5)	1653 (9.6)	145 (5.8)	67 (5.0)	78 (6.6)
Second	3206 (8.9)	1669 (8.9)	1537 (8.9)	193 (7.7)	107 (8.1)	86 (7.2)
Third	3165 (8.8)	1645 (8.8)	1520 (8.8)	211 (8.4)	113 (8.5)	98 (8.2)
Fourth	3188 (8.9)	1667 (8.9)	1521 (8.8)	214 (8.5)	119 (9.0)	95 (8.0)
Fifth	3461 (9.6)	1773 (9.4)	1688 (9.8)	217 (8.6)	118 (8.9)	99 (8.3)
Sixth	3472 (9.6)	1817 (9.7)	1655 (9.6)	244 (9.7)	122 (9.2)	122 (10.2)
Seventh	3629 (10.1)	1889 (10.1)	1740 (10.1)	263 (10.4)	138 (10.4)	125 (10.5)
Eighth	3721 (10.3)	1943 (10.3)	1778 (10.3)	266 (10.6)	136 (10.2)	130 (10.9)
Ninth	4065 (11.3)	2085 (11.1)	1980 (11.5)	323 (12.8)	166 (12.5)	157 (13.2)
Tenth	4675 (13.0)	2509 (13.4)	2166 (12.6)	441 (17.5)	240 (18.1)	201 (16.9)
Height velocity, mean (SD), cm/mo						
1 mo	2.3 (0.2)	2.4 (0.2)	2.2 (0.2)	2.4 (0.2)	2.4 (0.2)	2.3 (0.2)
6 mo	2.4 (0.2)	2.4 (0.2)	2.4 (0.2)	2.4 (0.2)	2.4 (0.2)	2.4 (0.2)
12 mo	2.3 (0.2)	2.3 (0.2)	2.4 (0.2)	2.4 (0.2)	2.4 (0.2)	2.5 (0.2)
24 mo	2.3 (0.2)	2.3 (0.2)	2.3 (0.2)	2.3 (0.2)	2.3 (0.2)	2.3 (0.2)
Weight velocity, mean (SD), g/mo						
1 mo	595 (107)	649 (94)	535 (87)	686 (138)	739 (128)	626 (124)
6 mo	659 (114)	673 (116)	643 (110)	587 (126)	591 (128)	583 (124)
12 mo	558 (111)	558 (112)	558 (108)	513 (120)	511 (124)	514 (115)
24 mo	546 (126)	539 (125)	554 (126)	561 (133)	563 (136)	560 (129)
Peak BMI, mean (SD)^e^	17.5 (1.2)	17.7 (1.2)	17.3 (1.2)	17.6 (1.3)	17.9 (1.3)	17.4 (1.3)
Age at peak BMI, mean (SD), mo	10.2 (2.5)	10.0 (2.5)	10.4 (2.6)	7.4 (2.4)	7.1 (2.2)	7.8 (2.4)
Rebound BMI, mean (SD)^e^	15.5 (1.2)	15.6 (1.2)	15.5 (1.3)	15.5 (1.3)	15.6 (1.3)	15.3 (1.3)
Age at rebound BMI, mean (SD), y	5.2 (1.5)	5.4 (1.4)	5.0 (1.6)	5.1 (1.5)	5.2 (1.4)	5.0 (1.6)
Overweight or obesity at 10 y						
No	31711 (88.0)	16679 (88.8)	15032 (87.2)	2099 (83.4)	1081 (81.5)	1018 (85.5)
Yes	4307 (12.0)	2101 (11.2)	2206 (12.8)	418 (16.6)	245 (18.5)	173 (14.5)

Abbreviation: BMI, body mass index.

^a^ The discovery cohort was born between 1991 and 2011.

^b^ The replication cohort was born between 1999 and 2010.

^c^ Birth weight was standardized for sex and gestational age using International Fetal and Newborn Growth Consortium for 21st Century standards.

^d^ See the Methods for decile definitions.

^e^ BMI is calculated as weight in kilograms divided by height in meters squared.

### Association With Infant Height and Weight Growth Velocity

**Figure 2.**
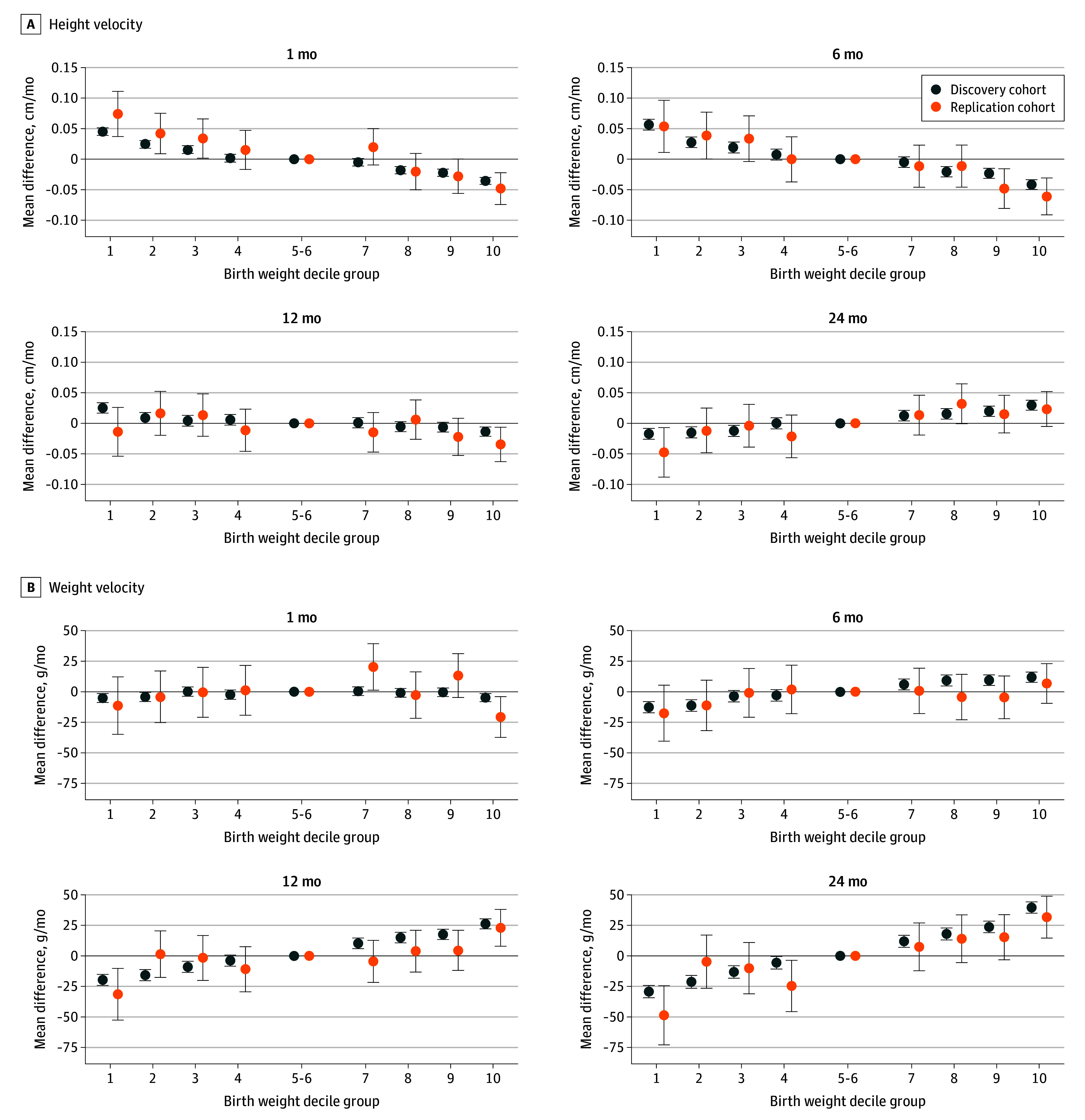
Dot Plots of Mean Difference in Infant Height and Weight Growth Velocity at Age 1, 6, 12, and 24 Months Figure shows mean differences (points) and 95% CIs (vertical bars) in infant height and weight growth velocity at ages 1, 6, 12, and 24 months for each birth weight decile group (vs fifth to sixth decile group). Models were fitted separately in the discovery and replication cohort and adjusted for sex and cohort. See the Methods for decile definitions. Numerical results are in eTable 2 in Supplement 1. To aid interpretation of differences, the estimated means for the reference (fifth to sixth decile) group are as follows: for height velocity at 1, 6, 12, and 24 months, 2.3, 2.4, 2.3, and 2.3 cm per month (discovery cohort), and 2.4, 2.4, 2.4, and 2.3 cm per month (replication cohort); for weight velocity at 1, 6, 12, and 24 months, 591, 657, 554, and 543 g per month (discovery cohort), and 686, 580, 501, and 553 g per month (replication cohort).

In the discovery cohort, when compared with the reference birth weight group (deciles 5-6), mean height velocity at age 1 month was higher for the 3 lowest birth weight groups, lower for the 3 highest groups, and similar for the 2 adjacent middle groups (Figure 2). This association persisted at age 6 months but was reduced at age 12 months. At 24 months, the direction of association had reversed, with height velocity becoming lower in the lowest 3 groups and higher in the highest 3 groups. For example, mean differences in height velocity for the second (vs fifth to sixth) birth weight decile group were 0.03 cm per month (95% CI, 0.02 to 0.04 cm per month) at age 6 months and −0.01 cm per month (95% CI, −0.02 to −0.01 cm per month) at age 24 months; mean differences for the ninth (vs fifth to sixth) birth weight decile group were −0.02 cm per month (95% CI, −0.03 to −0.01 cm per month) at age 6 months and 0.02 cm per month (95% CI, 0.01 to 0.03 cm per month) at age 24 months.

For weight velocity, associations were negligible at age 1 month but increased and remained directionally consistent with increasing age (Figure 2). Compared with the reference group, mean weight velocity at 6, 12, and 24 months was lower in all 4 lower birth weight groups and higher in all 4 higher groups. For example, mean differences in weight velocity at age 12 months were −15.8 g per month (95% CI, −20.4 to −11.2 g per month) for the second birth weight decile group and 17.5 g per month (95% CI, 13.3 to 21.8 g per month) for the ninth birth weight decile group (vs the fifth to sixth decile group). Replication cohort results were consistent with estimates for both height and weight velocity at all ages (Figure 2; eTable 2 in Supplement 1).

### Association With Height and Weight Growth From 1 Month to 5 Years

**Figure 3.**
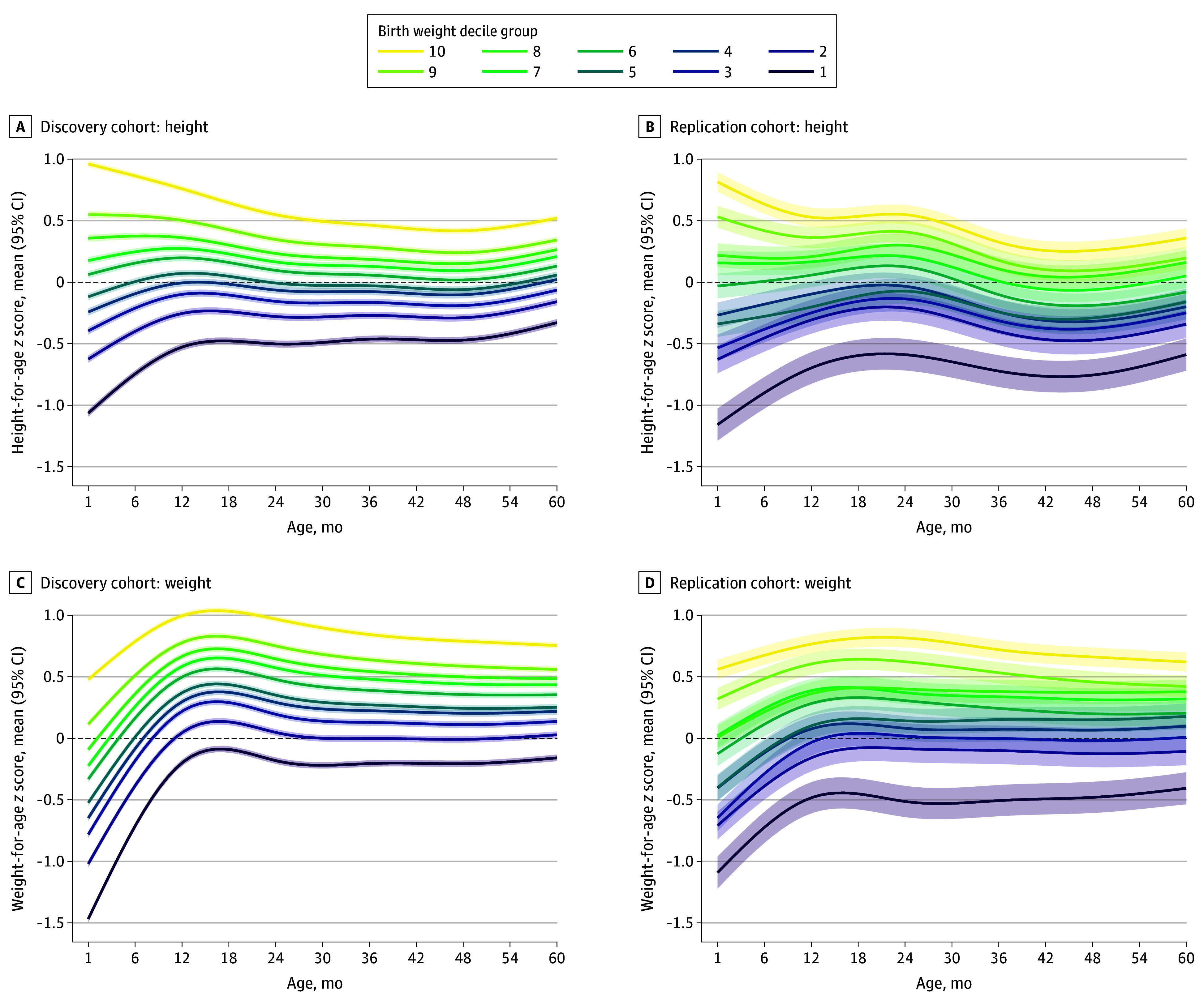
Line Graphs of Mean Height and Weight *z* Score Trajectory From Age 1 Month to 5 Years Figure shows estimated mean (lines) and 95% CIs (bands) for World Health Organization height-for-age and weight-for-age *z* score trajectory from 1 month to 5 years for each birth weight decile group (see the Methods for decile definitions). Models were fitted separately in the discovery and replication cohort. Models were adjusted for sex and cohort and included a natural spline for age plus its interaction with birth weight.

In the discovery cohort, mean height and weight *z* scores were higher in higher birth weight decile groups. The magnitude of the differences between birth weight groups was largest at the earliest age and progressively attenuated with age (Figure 3). For height, children in the lowest 3 and highest 4 birth weight groups remained below and above WHO average height up to age 5 years, respectively. For weight, only the lowest birth weight group and the highest 2 groups respectively remained below and above WHO average. The replication cohort showed consistent results for both height and weight.

### Association With Infant Peak BMI and Childhood Rebound BMI

**Figure 4.**
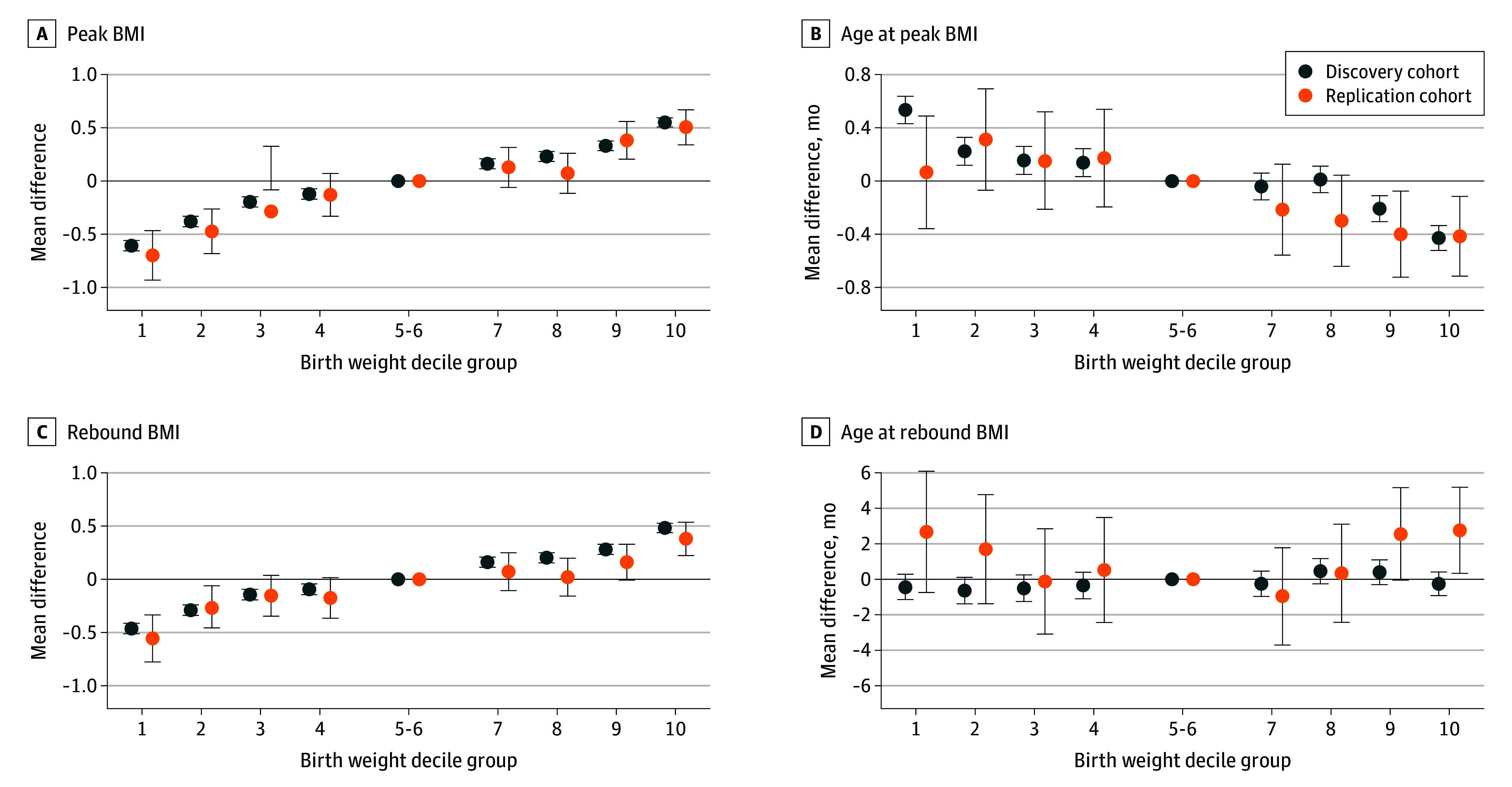
Dot Plots of Mean Difference in Magnitude and Timing of Infant Peak Body Mass Index (BMI) and Childhood Rebound BMI Figure shows mean differences (points) and 95% CIs (vertical bars) in BMI (calculated as weight in kilograms divided by height in meters squared) and age at infant peak BMI and childhood rebound BMI for each birth weight decile group (vs fifth to sixth decile group). Models were fitted separately in the discovery and replication cohort and adjusted for sex and cohort. See the Methods for decile definitions. Numerical results are presented in eTable 2 in Supplement 1. To aid interpretation of differences, the estimated means for the reference (fifth to sixth decile) group are as follows: peak BMI, 17.4 for the discovery cohort and 17.5 for the replication cohort); age at peak BMI, 10.0 months for the discovery cohort and 7.4 months for the replication cohort; rebound BMI, 15.4 for the discovery cohort) and 15.3 for the replication cohort; and age at rebound BMI, 61.7 months for the discovery cohort and 60.4 months for the replication cohort.

In the discovery cohort, when compared with the reference birth weight group (deciles 5-6), peak BMI and rebound BMI were lower in all 4 lower birth weight groups and higher in all 4 higher groups (Figure 4). For example, mean differences in peak BMI were −0.38 (95% CI, −0.43 to −0.33) for the second birth weight decile group and 0.33 (95% CI, 0.29 to 0.38) for the ninth birth weight decile groups (vs the fifth to sixth decile group). Compared with the reference group, mean age at peak BMI was higher in all 4 lower birth weight groups and lower for the highest 2 groups (Figure 4). For example, mean differences in age at peak BMI were 0.22 months (95% CI, 0.12 to 0.33 months) for the second birth weight decile group and −0.21 months (95% CI, −0.30 to −0.11 months) for the ninth birth weight decile groups (vs the fifth to sixth decile group). No clear difference in age at rebound BMI was found. The replication cohort showed results consistent with discovery cohort estimates for peak BMI, rebound BMI, and age at peak BMI, and a U-shaped association with age at rebound BMI was found, indicating higher mean age at rebound BMI in the 2 lowest and highest groups vs the reference (Figure 2; eTable 2 in Supplement 1).

### Association With Overweight or Obesity at Age 10 Years

In the discovery cohort, compared with the reference birth weight group (deciles 5-6), the risk of overweight or obesity at age 10 years was lower for the 2 lowest birth weight groups and was higher in all 4 higher birth weight groups (eTable 2 in Supplement 1). For example, risk ratios for overweight or obesity at 10 years were 0.86 (95% CI, 0.76 to 0.97) for the second birth weight decile group and 1.25 (95% CI, 1.13 to 1.38) for the ninth birth weight decile group (vs the fifth to sixth deciles). Replication cohort results were consistent with these estimates (eTable 2 in Supplement 1).

### Additional Discovery Cohort Results

Differences in height and weight *z* score trajectories by birth weight decile appeared larger in girls vs boys (eFigure 4 in Supplement 1), with no evidence of sex differences in associations with any other outcomes. Using birth weight deciles provided only modest gains in estimation performance over conventional groups, with the largest gains for weight and height *z* score trajectories (increase in *R^2^*, 4.3% and 3.8%, respectively), followed by peak BMI (2.5% increase) and rebound BMI (1.7% increase). Comparing linear and nonlinear terms for birth weight *z* score identified linear associations with most outcomes, subtle nonlinear associations with height velocity at age 1 and 6 months, and an inverse U-shaped association with weight velocity at age 1 month (eFigure 5 in Supplement 1). Results were similar after excluding preterm and postterm births (eTable 3 in Supplement 1), and after adjustment for confounders (eTables 4 and 5 in Supplement 1).

## Discussion

In this cohort study, we used data from more than 36 000 European and more than 2500 non-European singletons born 1991 to 2011 to quantify and replicate associations of birth weight–for–gestational age percentiles with infant and child growth features. Compared with middle (fifth to sixth decile) groups, higher birth weight groups showed lower early infant height velocity, which reversed by 24 months, and increasingly higher weight velocity through infancy; lower birth weight groups showed the opposite pattern. Infants in the lowest birth weight group remained below WHO average height and weight up to age 5 years, whereas infants in the highest group exceeded them. Compared with the middle groups, higher birth weight groups showed higher peak and rebound BMI, earlier peak (but not rebound) BMI, and higher risk of overweight or obesity at 10 years, with lower birth weight groups showing the opposite pattern. Results were replicated in an independent cohort, consistent in male and female children, and mainly linear in nature.

Our findings expand on previous studies^9,10,11,32,39,40,41,42,43,44,45^ that examined associations between conventional birth weight–for–gestational age groups or continuous birth weight scores and child growth outcomes. Our discovery analysis results in European birth cohorts were replicated in our pooled US and Singapore birth cohorts and agree with studies across diverse populations.^9,10,11,32,39,40,41,42,43,44,45,46^ The predominantly linear associations across the birth weight range challenge the assumption of conventional categories that all AGA infants share a uniform risk profile. Differences in growth outcomes (vs middle of the percentile distribution) were greatest at lowest and highest birth weight percentiles, which aligns with evidence of increased morbidity risk at both ends of the percentile distribution, and increased mortality rate at lowest percentiles.^47,48^ Although neonatal mortality rate is typically lowest at higher birth weight percentiles,^12,47,48^ our results suggest that infants at higher birth weight percentiles might also carry incrementally higher risk for obesogenic growth patterns.^8,49^

Clinicians should therefore be aware that infants at upper birth weight percentiles, even below the LGA threshold, may still be at elevated risk for adverse growth patterns and overweight, and may benefit from targeted postnatal monitoring. Our findings also suggest that birth weight decile might be a useful prognostic indicator, including across diverse settings and both sexes given our largely consistent findings. However, its estimation advantage over conventional groups was modest, indicating that traditional groupings (LGA, SGA, and AGA) remain robust, pragmatic tools for detecting postnatal growth differences.

Infant and childhood growth patterns can be conceptualized as a continuation of intrauterine trajectories linked to birth anthropometrics. Genetic factors are likely to explain some of our findings, particularly for outcomes beyond infancy where perinatal influences diminish,^50,51,52,53^ and future studies could explore the distinct maternal and paternal genetic contributions to fetal and postnatal growth. Differences in intrauterine environments may also play a role^37,38^; for example, faster linear growth in early infancy in the lowest birth weight group could reflect compensatory catch-up after intrauterine growth restriction.^54,55^ That associations remained robust to adjustment for confounders, including both parents’ BMI, supports this hypothesis, but further study and triangulation is needed. Finally, the narrowing of *z* score trajectory differences between birth weight groups with age is consistent with regression to the mean,^56^ potentially reflecting transient in utero effects and progressive realignment with genetically predetermined growth trajectories as children age.

### Limitations

This study has limitations that should be mentioned. Pooling multiple European birth cohorts provided a large sample size for granular analyses across the birth weight range, and using a geographically distinct replication cohort helps improve generalizability. However, combining different cohorts may obscure underlying cohort, country, and secular differences, including in fetal and postnatal growth patterns. Therefore, the true magnitude of associations may vary across local populations. Participants were born 15 to 35 years before the present day in high-income countries in Europe, US, and Singapore, and so findings may not generalize to more recent birth cohorts or lower-income settings.

INTERGROWTH-21st standards were used to ensure consistent methods across cohorts; however, these standards are known to underclassify SGA and overclassify LGA,^57^ which likely explains our skewed decile distribution and may limit generalizability of our cutoffs to local settings. Birth weight was not standardized for parental anthropometry; however, using more customized birth weight percentiles may not provide additional estimation benefits beyond noncustomized charts.^58^ Still, we are unable to distinguish constitutionally small and large births from pathological growth constraint or overgrowth, respectively. Moreover, because our analysis relies solely on birth weight, it does not account for variation in neonatal length or body proportionality and the resulting inherent heterogeneity within birth weight groups. Furthermore, we did not include fetal measurements and so were unable to account for specific intrauterine trajectories preceding birth weight.

We excluded children with missing birth weight or gestational age data, children with fewer than 2 repeated growth measurements, and those with no identifiable BMI peak or rebound, which may have reduced precision of our estimates. Consistent with prior studies, we used a conventional adult BMI formulation, which may not be optimal for children.^59^ Although peak and rebound BMI, and their timing, reflect developmental dynamics underlying obesity risk and are, therefore, of public health relevance, their identification relies on longitudinal growth measurements that may not always readily available in clinical settings. In addition, because BMI is an indirect marker of adiposity, we are unable to distinguish between fat mass and lean mass at the BMI peak and rebound.^60^

## Conclusions

In this cohort study of 38 535 singletons, birth weight–for–gestational age decile groups were associated with infant and childhood growth dynamics and overweight or obesity. Although the benefits of birth weight decile group analysis over conventional groups were modest, our findings suggest that the risk of obesogenic growth may increase incrementally across the AGA range and is not confined to the extremes of the percentile distribution. The robustness of these mostly linear associations across populations and sex suggest that birth weight decile group may be a useful supplementary screening tool, potentially identifying at-risk infants missed by traditional cutoffs. The findings also suggest the potential value of early intervention, including antenatally, to improve early life growth. Future research should develop postnatal growth prediction models combining birth weight percentile with other established early life factors.

## Notes

Supplement 1. eMethods. eReferences eTable 1. Birth and infant/child growth outcomes in each individual birth cohort eTable 2. Sex and cohort-adjusted mean differences in infant/child growth outcomes in the Discovery and Replication cohorts eTable 3. Sex and cohort-adjusted mean differences in infant/child growth outcomes in the Discovery cohort, after excluding preterm and post-term births eTable 4. Parental pregnancy-related factors in the Discovery cohort eTable 5. Confounder-adjusted mean differences in infant/child growth outcomes in the Discovery cohort eFigure 1. Observed height, weight, and BMI in the Discovery and Replication cohorts eFigure 2. Predicted individual-specific growth trajectories in the Discovery and Replication cohorts eFigure 3. Correlations between infant/child growth outcomes in Discovery and Replication cohorts eFigure 4. Mean height and weight Z score trajectory from age 1 month to 5 years, presented by sex in the Discovery cohort eFigure 5. Linear and nonlinear associations between continuous birth weight Z-score and infant/child growth outcomes in the Discovery cohort eAppendix. Cohort-specific acknowledgements and funding

Supplement 2. Data Sharing Statement
