# Error in Figure 2 Caption

**DOI:** 10.1001/jamanetworkopen.2026.31818

**Published:** 2026-08-10

In the Original Investigation titled “Birth Weight Percentiles and Infant and Child Growth Dynamics,”^1^ published July 8, 2026, the units of measure for grams and centimeters were reversed in the Figure 2 caption. This article has been corrected.^1^
