# Supplementary material for: Birth Weight Percentiles and Infant and Child Growth Dynamics
Source: JAMA Netw Open. 2026 Jul 8;9(7):e2622218. doi: 10.1001/jamanetworkopen.2026.22218 (PMC13347239; doi:10.1001/jamanetworkopen.2026.22218)
Supplement: Supplement 2. — Data Sharing Statement [file jamanetwopen-e2622218-s002.pdf]

# Data Sharing Statement

Hernandez. Birth Weight Percentiles and Infant and Child Growth Dynamics. *JAMA Netw Open*. Published July 08, 2026. doi:10.1001/jamanetworkopen.2026.22218

**Data**
**Data available:** No

**Additional Information**
**Explanation for why data not available:** The informed consent obtained from each cohort study's participants does not allow the data to be made available through any third party maintained public repository. The data used in this study can be made available to bona fide researchers on request to each cohort study, who will then release their data.
